# Chromosome-level genome assembly of the flower thrips *Frankliniella intonsa*

**DOI:** 10.1038/s41597-023-02770-3

**Published:** 2023-11-30

**Authors:** Zhijun Zhang, Jiandong Bao, Qizhang Chen, Jianyun He, Xiaowei Li, Jiahui Zhang, Zhixing Liu, Yixuan Wu, Xuesheng Li, Yunsheng Wang, Yaobin Lu

**Affiliations:** 1 State Key Laboratory for Managing Biotic and Chemical Threats to the Quality and Safety of Agro-Products, Institute of Plant Protection and Microbiology, Zhejiang Academy of Agricultural Sciences, Hangzhou, 310021 China; 2 Hunan Provincial Key Laboratory for Biology and Control of Plant Diseases and Insect Pests, Hunan Agricultural University, Changsha, 410125 China; 3 Guangxi key laboratory of Agric-Environment and Agric-products Safety, Guangxi University, Nanning, Guangxi 530004 China

**Keywords:** Agricultural genetics, Entomology, Innate immunity

## Abstract

As an economically important insect pest, the flower thrips *Frankliniella intonsa* (Trybom) causes great damage to host plants by directly feeding and indirectly transmitting various pathogenic viruses. The lack of a well-assembled genomic resource has hindered our understanding of the genetic basis and evolution of *F. intonsa*. In this study, we used Oxford Nanopore Technology (ONT) long reads and High-through chromosome conformation capture (Hi-C) linked reads to construct a high-quality reference genome assembly of *F. intonsa*, with a total size of 225.5 Mb and a contig N50 of 3.37 Mb. By performing the Hi-C analysis, we anchored 91.68% of the contigs into 15 pseudochromosomes. Genomic annotation uncovered 17,581 protein-coding genes and identified 20.09% of the sequences as repeat elements. BUSCO analysis estimated over 98% of genome completeness. Our study is at the first time to report the chromosome-scale genome for the species of the genus *Frankliniella*. It provides a valuable genomic resource for further biological research and pest management of the thrips.

## Background & Summary

The flower thrips, *Frankliniella intonsa* (Trybom) (Thysanoptera: Thripidae), is a small-sized insect pest, well known for feeding and dwelling on the flower of host plants. It is widely distributed in the world including Europe, Asia, Oceania, and North America^1,2^, and becoming the dominant thrips species in several areas of China^3,4^. By rapid development and both sexual and parthenogenetic reproductive modes, *F. intonsa* is able to cause severe damage to various commercial crops, such as cowpea, eggplant^5–9^, and rapidly accumulated resistance to insecticides like spinosad^10^. In addition to direct damage to the leaves, flowers and fruits, *F. intonsa* is also capable of transmitting a variety of plant pathogenic viruses, such as Tomato spotted wilt orthotospovirus (TSWV) and Chrysanthemum stem necrosis virus (CSNV) to host plants^11,12^, resulting in destructive damage and huge economic losses every year. Interestingly, we found that the endosymbiont *Wolbachia* is dwelling in *F. intonsa*, but is absent in the sibling species *F. occidentalis* (unpublished data). High-quality genomic resources are urgently needed to elucidate the key genetic mechanisms of flower thrips like virus transmission, pheromone biosynthesis, insecticide resistance and bacterial mutualism.To date, despite the large number of species in the family Thripidae (true thrips), only five species have publicly available genomes, including a scaffold-level genome assembly of western flower thrips (*F. occidentalis*, 415.8 Mb)^13^, a chromosome-level genome assembly of melon thrips (*Thrips palmi*)^14^, a scaffold-level genome assembly of tobacco thrips (*F. fusca*, 370 Mb)^15^, and two chromosome-level genome assembly of bean blossom thrips (*Megalurothrips usitatus*) reported by Ma *et al*.^16^ (238.14 Mb) and our group^17^ (247.82 Mb), respectively. However, there is no reported genome assembly for *F. intonsa*, nor chromosome-level genome for the *Frankliniella* genus species. In this study, we report a high-quality chromosome-level genome assembly of *F. intonsa* using an integrated sequencing strategy including ONT, Illumina, and Hi-C. Our research provides valuable resources for studying the evolutionary genetics and molecular basis of *F. intonsa*.

**Fig. 1:**
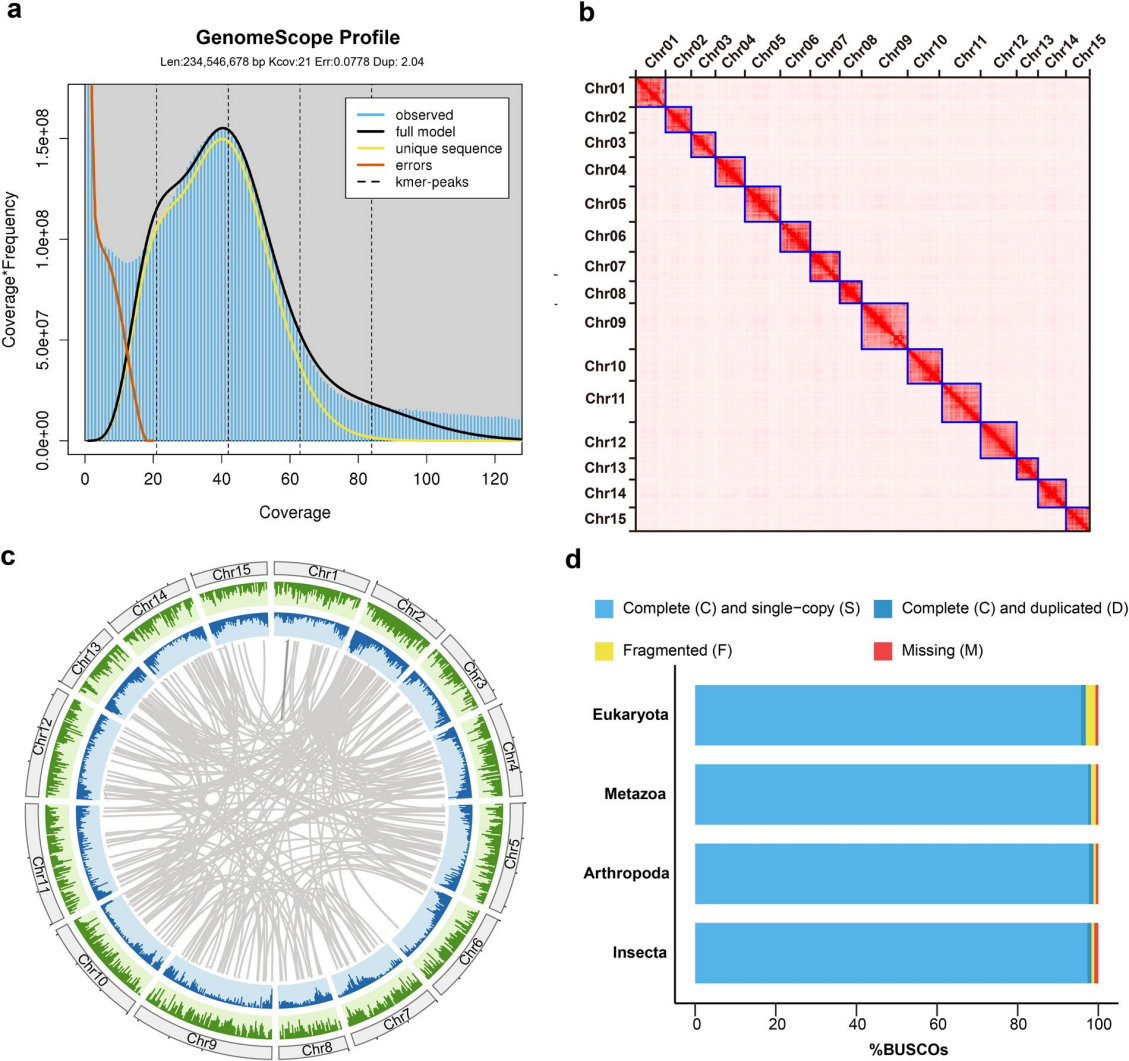
Characteristics of the *F. intonsa* genome. (**a**) Estimation of genome size based on NGS reads using 21-kmer analysis. (**b**) The heatmap represents 15 pseudochromosomes of the *F. intonsa* genome. (**c**) The cirsos plot describes the genomic characteristics of *F. intonsa*, including the chromosomes (the outer circle), gene density (green cycle), repeat density (blue circle), and paralogous genes (line plot). (**d**) BUSCO analysis based on the Eukaryota, Metazoa, Arthropoda and Insecta (odb_10) data sets.

To obtain a high-quality genome assembly of *F. intonsa*, a total of 31.63 Gb ONT long reads (~124-fold coverage) and 15.71 Gb NGS short reads (~62-fold coverage, 2 × 150 bp) were generated. Using the integrated sequencing data, we obtained a contig-level genome with a total size of 225.5 Mb, consisting of 405 contigs with N50 length of 3.37 Mb and N90 length of 161 Kb. (Table 1). The total length of the genome assembly is similar to the estimated genome size (approximately 234.5 Mb) based on 21-mer depth analysis (Fig. 1a). The total GC content is 52.73%, which is comparable to the other Thripidae species^13–17^. To improve the continuity of the genome assembly, we exploited 41.97 Gb (~165-fold coverage) Hi-C data, which generated about 57 million Hi-C contacts, to concatenate the contigs. Approximately 91.68% of the contig sequence was successfully anchored into 15 pseudochromosomes ranging from 9.78 Mb to 20.82 Mb (Fig. 1b,c). We further performed BUSCO analysis to assess the completeness of the genome assembly based on four categories of datasets, including Eukaryota, Metazoa, Arthropoda and Insecta (odb_10). As a result, 96.9% conserved Eukaryotic genes and more than 98% of the core genes in the other three datasets were identified, strongly suggesting a high level of completeness of the *F. intonsa* genome assembly (Fig. 1d).

**Table 1:** Statistics of *F. intonsa* reference genome.

Genome features	*F. intonsa*
Total,size of contig assembly (Mb)	225.5
Number of contig	405
Contig N50 (bp)	3,373,233
Contig N90 (bp)	161,309
Number of pseudochromosomes	15
Scaffold N50 (bp)	13,489,000
Scaffold N90 (bp)	9,782,500
GC content	52.73%
Repeat content	20.09%
Number of protein coding genes	17,581
Average gene length (bp)	3,293
Average exon number Per gene	6.28

Using multiple repeat annotation software, we constructed a repeat library containing 1,347 repeat consensus sequences in the *F. intonsa* genome. We then performed a genome-wide scan of repeat-associated regions based on the repeat library. As a result, we annotated approximately 20.09% repeat regions in the *F. intonsa* genome (Table 1). Remarkably, the repeat content in the *F. intonsa* genome is significantly higher than in the other published genomes of Thripidae species (*F. occidentalis* (9.86%), *M. usitatus* (15.05%), *T. palmi* (6.45%)), suggesting the amplification of repeat elements in the *F. intonsa* genome^13–17^.

**Fig. 2:**
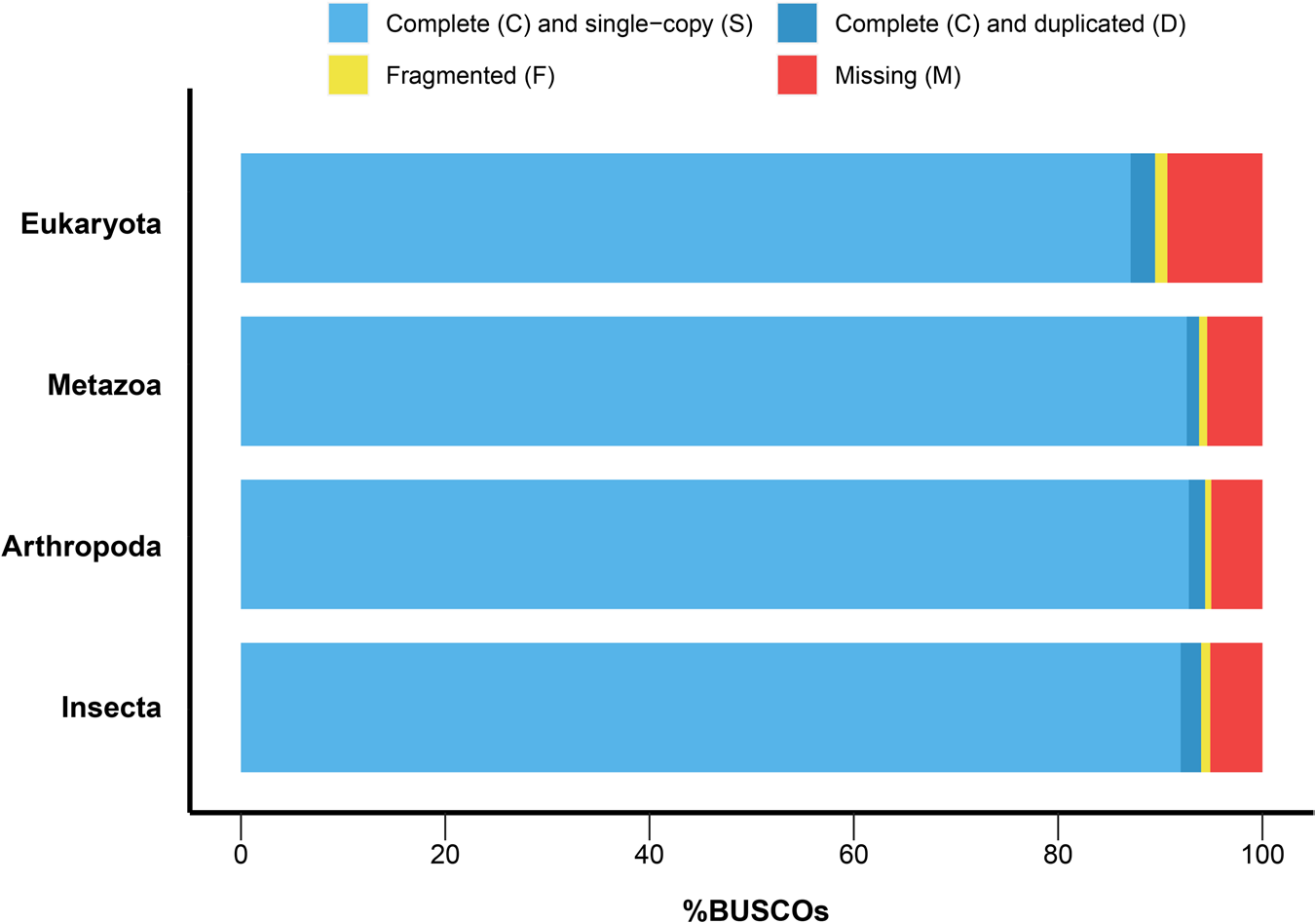
BUSCO analysis of the gene models based on the Eukaryota, Metazoa, Arthropoda and Insecta (odb_10) data sets.

**Fig. 3:**
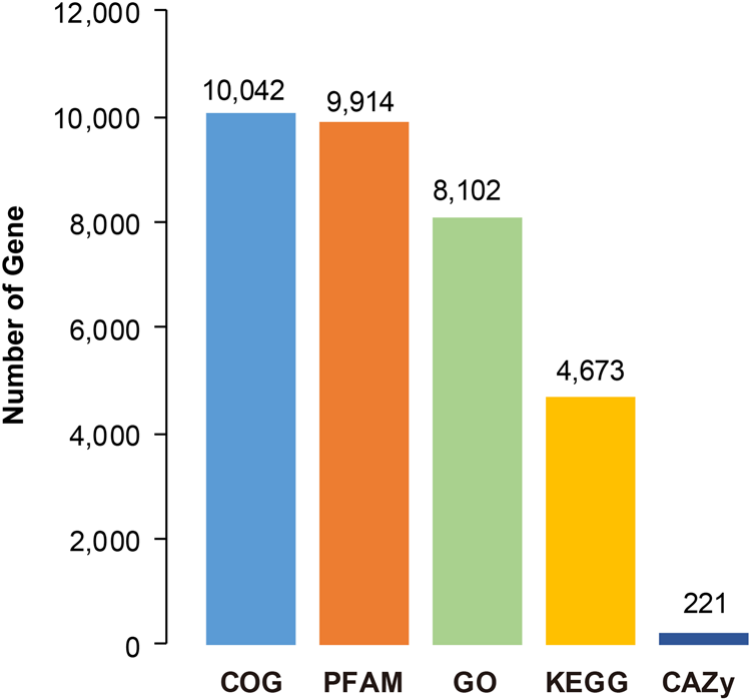
The number of genes annotated with COG, PFAM, GO, KEGG and CAZy databases.

A combined approach of ab initio prediction, homolog-based prediction, and transcript-based prediction was used to predict gene structure in the *F. intonsa* genome. This resulted in a total of 17,581 protein-coding genes, which is comparable to other Thripidae species. BUSCO analysis using the gene model showed that 89.5%, 93.8%, 94.4% and 94.0% of the core genes from the Eukaryota, Metazoa, Arthropoda and Insecta datasets, respectively, were complete, indicating a high level of completeness and credibility of the gene prediction results (Fig. 2). Then, we functionally annotated the protein-coding genes based on five major databases, including Gene Ontology (GO), Kyoto Encyclopedia of Genes and Genomes (KEGG), Pfam, Clusters of Orthologous Genes (COG) and the Carbohydrate-Active enZYmes (CAZy). More than half of the genes (58.42%, 10,272/17,581) were well annotated with at least one functional result (Fig. 3).

## Methods

### Sampling, extraction, and sequencing

*F. intonsa* populations were collected on pepper (*Capsicum annuum L*.) from Jiaxing (30.75° N, 120.79° E), Zhejiang, China, in 2017, and reared on fresh bean*, Phaseolus vulgaris*, in a climate-controlled chamber (25 ± 1 °C, 16 L). Approximately 200 adult *F. intonsa* samples from the laboratory population with mixed ages were decontaminated by immersion in 1% sodium hypochlorite solution (Gaide chemical, Hangzhou, Zhejiang, China) for 5 min, and followed by rinsing with sterile water and then immersion in 70% ethanol and finally rinsing with sterile water again. Samples were snap frozen in liquid nitrogen and stored at −80 °C.

Genomic DNA and mRNA were extracted and purified using QIAGEN DNA/RNA tissue kit (QIAGEN 69506/73404, Hilden, Germany), and prepared for sequencing libraries according to the manufacturer’s instructions for sequencing technology (Nextomics Biosciences Co., Ltd, Wuhan, China). Long DNA fragments were sequenced on the Oxford Nanopore PromethION platform, and short-read sequencing, Hi-C sequencing and RNA-seq were performed on the Illumina NovaSeq. 6000 platform (Table S1).

### Genome assembly, scaffolding, completeness evaluation

The raw Oxford nanopore sequencing technology (ONT) reads were quality controlled using LongQC (v1.2.0c)^18^ to remove low-quality, barcode and adapter sequences. The clean ONT reads were then used for *de novo* assembly using NextDenovo (v2.5.2)^19^ with an expected genome size of 250 Mb, and the draft contig assembly was polished by NextPolish (v1.4.1)^20^ with default parameters using both of the clean ONT reads and NGS short reads. To obtain chromosome-level data, we exploited Hi-C sequencing data to concatenate the contigs based on chromosomal interaction signals. Bowtie2 (v2.5.1)^21^ and 3D-DNA (v180114)^22^ were used for scaffolding, and Juicebox (v2.20.00)^23^ was then used for manual scaffold correction. To assess the completeness of genome assembly, we performed BUSCO (v5.4.4)^24^ analysis with the genome model by using the Eukaryota, Metazoa, Arthropoda and Insecta (odb10) datasets (https://busco-data.ezlab.org/v5/data/lineages/). Clean NGS and ONT reads were realigned to the genome assembly using bwa-mem2 (v2.2.1)^25^ and minimap2 (v2.26)^26^, respectively.

### Repeat annotation

Repeatmodeler (v2.0.3)^27^ in combination with LTR_finder (v1.07)^28^, LTRharvest (v1.6.2)^29^ and LTR_retriever (v2.9.0)^30^ were used to identify repeat elements, including LTR retrotransposons, non-LTR retrotransposons, DNA transposons and microsatellites. The *de novo* repeat library was then used as a seed for RepeatMasker (v4.1.0)^31^ to find and mask all repeat regions in the final genome assembly.

### Gene structural and functional annotation

We exploited several software programs including Funannotate (v1.5.3)^32^, Fgenesh^33^, Exonerate (v2.2.0)^34^, and Trinity (v2.11.0)^35^ to predict gene structure. Finally, EVidenceModeler (v2.0.0)^36^ was used to combine *de novo*, homolog-based and transcriptome-based gene predictions into a final gene structure dataset. Two software, eggNOG-mapper (v2.1.9)^37^ and Interproscan (v5.62-94.0)^38^, in combination with five databases including KOG, KEGG, CAZys, Pfam and Gene Ontology (GO) were used to functionally annotate the genes.

## Data Records

The genome sequence and annotation data were deposited in the Genome Warehouse (GWH, https://ngdc.cncb.ac.cn/gwh) at the National Genomic Data Center (NGDC)^39^, under the accession number of GWHDOOB00000000. Raw data from Nanopore (CRR824223^40^), Illumina (CRR825014^41^, CRR825015^42^) and Hi-C (CRR824225^43^) genome sequencing and RNA-seq (CRR824226^44^) were deposited in the Genome Sequence Archive^45^ (GSA, https://ngdc.cncb.ac.cn/gsa) at the NGDC. All data were related to the BioProject PRJCA018338. The genome sequence and raw reads were also deposited at WGS (JAWJED000000000.1^46^) and GSA (SRR26384730^47^ for Nanopore, SRR26384729^48^ for Illumina, SRR26384728^49^ for Hi-C, and SRR26384727^50^ for RNA-seq data) at NCBI, respectively, under BioProject PRJNA1027977.

## Technical Validation

Two different strategies were used to evaluate the completeness and accuracy of the *F. intonsa* genome. First, BUSCO analysis based on the Aukaryota, Metazoa, Arthropoda and Insecta (odb_10) datasets revealed that 96.9%, 98.2%, 98.8% and 98.3% of the core genes were successfully identified as complete. Second, we re-aligned the NGS, ONT and RNA-seq reads to the *F. intonsa* genome with the mapping rates of 92.80%, 90.48% and 88.63%, respectively. For evaluation of gene prediction completeness and accuracy, we performed BUSCO analysis based on the Eukaryota, Metazoa, Arthropoda and Insecta (odb_10) datasets, which resulted in 89.5%, 93.8%, 94.4% and 94.0% of completeness, respectively.

### Supplementary information

Supplemental Information

## Data Availability

No specific script was used in this work. The codes and pipelines used for data processing were all executed according to the manual and protocols of the corresponding bioinformatics softwares (detail parameters see Table S2).
