# Supplementary material for: Chromosome-level genome assembly of the flower thrips Frankliniella intonsa
Source: Sci Data. 2023 Nov 30;10:844. doi: 10.1038/s41597-023-02770-3 (PMC10689740; doi:10.1038/s41597-023-02770-3)
Supplement: Supplementary file 1 — Supplemental Information [file 41597_2023_2770_MOESM1_ESM.docx]

**Supplemental Information for:**

**Chromosome-level genome assembly of** **the flower thrips** ***Frankliniella intonsa***

**Zhijun Zhang ^1,^*, Jiandong Bao ^1^, Qizhang Chen ^1,3^, Jianyun He ^1^, Xiaowei Li ^1^, Jiahui Zhang ^1,2^, Zhixing Liu ^1^, Yixuan Wu ^1^, Xuesheng Li^3^, Yunsheng Wang ^2^, Yaobin Lu ^1,^***

1. State Key Laboratory for Managing Biotic and Chemical Threats to the Quality and Safety of Agro-Products, Institute of Plant Protection and Microbiology, Zhejiang Academy of Agricultural Sciences, Hangzhou 310021, China;

2. Hunan Provincial Key Laboratory for Biology and Control of Plant Diseases and Insect Pests, Hunan Agricultural University, Changsha 410125, China;

3. Guangxi key laboratory of Agric-Environment and Agric-products Safety, Guangxi University, Nanning, Guangxi 530004, China;

***** Correspondence: zhangzj@zaas.ac.cn (Z.Z.); lvyb@zaas.ac.cn (Y.L.);

**Table of Contents:**

| **Table S1** | Page 3 |
| --- | --- |
| **Table S2** | Page 4 |

**Table S1** Sequence reads used in this study

| **Reads type** | **Sequencing Platform** | **Size (Gb)** | **Coverage (×)** | **Application** | **NGDC GSA accessions** | **NGDC BioProject** | **NCBI SRA Accession** | **NCBI BioProject** |
| --- | --- | --- | --- | --- | --- | --- | --- | --- |
| ONT | Oxford Nanopore PromethION | 31.63 | 124 | Genome assembling | CRR824223 | PRJCA018338 | SRR26384729 | PRJNA1027977 |
| NGS | Illumina NovaSeq 6000 | 15.71 | 62 | Genome assemebly polishing | CRA011862 | PRJCA018338 | SRR26384730 | PRJNA1027977 |
| Hic | Illumina NovaSeq 6000 | 41.97 | 165 | Scaffolding to chromosome level | CRR824225 | PRJCA018338 | SRR26384728 | PRJNA1027977 |
| RNAseq | Illumina NovaSeq 6000 | 7.7 | 30 | Gene Annotation | CRR824226 | PRJCA018338 | SRR26384727 | PRJNA1027977 |

**Table S2** Software used for the analysis in this study

| **Software** | **Command line/online website** | **Usage** |
| --- | --- | --- |
| Juicer | juicer.sh -g Fint -z reference/genome.fa -y genome_DpnII.txt -p genome.chrom.sizes -s DpnII -D ./ | Mapping Hic data and generate Hic heat map |
| 3D-DNA | run-asm-pipeline.sh genome.fa merged_nodups.txt | Contig reassemble and scaffolding to Chromesome-level with Hic data |
| BUSCO | busco -m geno -i Fint.fa -l dataset_odb10 -c 6 --offline | Genome completeness evaluation |
| bwa-mem2 | bwa-mem2 mem -t 32 Fint_genome.fa NGS_1.fq.gz NGS_2.fq.gz \|samtools sort -@20 >NGS_sorted.bam | Mapping genomic NGS short reads |
| minimap2 | minimap2 -x map-ont -t 32 Fint_genome.fa ONT.fq.gz \|samtools sort -@20 >ONT_sorted.bam | Mapping genomic ONT long reads |
| samtools | for i in *.bam;do samtools flagstat -@20 $i >$i.stat;done | Simple statistics of mapped reads |
| Repeatmodeler | RepeatModeler -pa 50 -database Fint -engine rmblast -LTRStruct | Denovo repeat identification |
| RepeatMasker | RepeatMasker -lib Fint-families.fa Fint.fasta -pa 10 -nolow -no_is -norna -gff | Repeat masking |
| funannotate | funannotate predict -i genome.mask.fa -o output --name MusiPT_ -s "Megalurothrips usitatus" --protein_evidence uniprot_sprot.fasta --cpus 48 --max_intronlen 10000 --organism other --optimize_augustus | Genes structure annotation |
| eggNOG-mapper | http://eggnog-mapper.embl.de/ | Gene functional annotation |
| longQC | python longQC.py sampleqc -x ont-ligation -o output ONT.fq | Reads quality control |
| NextDenovo | nextDenovo run_fint.cfg | Denovo genome assembly |
| NextPolish | python nextPolish.py -g genome.ND.fa -t 5 --bam_lgs genome.lgs.bam -p 50 > genome.lgspolish.fa | Draft genome base-error correction |
| Bowtie2/Samtools | bowtie2 -t -p 4 -x ref --local -k 1 --no-unal -1 left.fq.gz -2 right.fq.gz \|samtools view -bS \|samtools sort -o out.bam | Mapping RNA-seq short reads |
| Exonerate | exonerate --model est2genome --percent 70 --dpmemory 32768 --showtargetgff 1 --showalignment 1 --maxintron 10000 cds.fasta Fint.fasta > out.exonerate.txt | Genes structure annotation with CDS sequence |
| Genomescope | http://qb.cshl.edu/genomescope/genomescope2.0/ | Genome size estimation |
